# Bis(5-methyl­pyrazine-2-carboxyl­ato)­diphenyl­tin(IV)

**DOI:** 10.1107/S1600536808016139

**Published:** 2008-06-07

**Authors:** Zhongjun Gao

**Affiliations:** a Department of Chemistry, Jining University, Shandong 273155, People’s Republic of China

## Abstract

In the mol­ecule of the title compound, [Sn(C_6_H_5_)_2_(C_6_H_5_N_2_O_2_)_2_], two O and one N atoms from the two 5-methyl­pyrazine-2-carboxyl­ate ligands and one C atom of a phenyl group form a distorted square-planar arrangement in the equatorial plane around the Sn atom, while the distorted octa­hedral coordination is completed by an N atom of one of the 5-methyl­pyrazine-2-carboxyl­ate ligands and a C atom of the other phenyl group in the axial positions. In the crystal structure, inter­molecular C—H⋯O hydrogen bonds link the mol­ecules into centrosymmetric dimers.

## Related literature

For general background, see: Gielen *et al.* (1988 ▶). For related literature, see: Vollano *et al.* (1984 ▶); Ma *et al.* (2004 ▶).
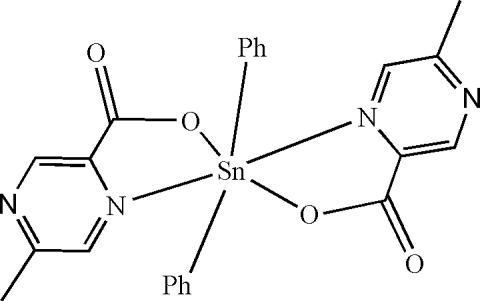


## Experimental

### 

#### Crystal data

[Sn(C_6_H_5_)_2_(C_6_H_5_N_2_O_2_)_2_]
*M* *_r_* = 547.13
Monoclinic, 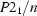

*a* = 12.030 (4) Å
*b* = 14.658 (5) Å
*c* = 13.409 (5) Å
β = 91.872 (4)°
*V* = 2363.2 (14) Å^3^
*Z* = 4
Mo *K*α radiation
μ = 1.12 mm^−1^
*T* = 298 (2) K
0.45 × 0.43 × 0.18 mm

#### Data collection

Bruker SMART CCD area-detector diffractometer
Absorption correction: multi-scan (*SADABS*; Sheldrick, 1996 ▶) *T*_min_ = 0.633, *T*_max_ = 0.824
12010 measured reflections
4166 independent reflections
2732 reflections with *I* > 2σ(*I*)
*R*_int_ = 0.039

#### Refinement

*R*[*F* ^2^ > 2σ(*F* ^2^)] = 0.035
*wR*(*F* ^2^) = 0.132
*S* = 1.05
4166 reflections
298 parameters
H-atom parameters constrained
Δρ_max_ = 0.56 e Å^−3^
Δρ_min_ = −0.46 e Å^−3^

### 

Data collection: *SMART* (Bruker, 1998 ▶); cell refinement: *SAINT* (Bruker, 1998 ▶); data reduction: *SAINT*; program(s) used to solve structure: *SHELXS97* (Sheldrick, 2008 ▶); program(s) used to refine structure: *SHELXL97* (Sheldrick, 2008 ▶); molecular graphics: *SHELXTL* (Sheldrick, 2008 ▶); software used to prepare material for publication: *SHELXTL*.

## Supplementary Material

Crystal structure: contains datablocks I, global. DOI: 10.1107/S1600536808016139/hk2458sup1.cif

Structure factors: contains datablocks I. DOI: 10.1107/S1600536808016139/hk2458Isup2.hkl

Additional supplementary materials:  crystallographic information; 3D view; checkCIF report

## Figures and Tables

**Table:** 

Sn1—O1	2.086 (4)
Sn1—O3	2.091 (4)
Sn1—C13	2.117 (5)
Sn1—C19	2.130 (6)
Sn1—N1	2.357 (4)
Sn1—N3	2.363 (5)

**Table:** 

O1—Sn1—O3	149.56 (15)
O1—Sn1—C13	97.14 (18)
O3—Sn1—C13	101.11 (18)
O1—Sn1—C19	101.5 (2)
O3—Sn1—C19	96.7 (2)
C13—Sn1—C19	105.6 (2)
O1—Sn1—N1	73.49 (15)
O3—Sn1—N1	82.40 (15)
C13—Sn1—N1	163.15 (18)
C19—Sn1—N1	90.17 (18)
O1—Sn1—N3	83.74 (16)
O3—Sn1—N3	73.16 (16)
C13—Sn1—N3	87.08 (18)
C19—Sn1—N3	165.3 (2)
N1—Sn1—N3	78.12 (15)

**Table 2:** Hydrogen-bond geometry (Å, °)

*D*—H⋯*A*	*D*—H	H⋯*A*	*D*⋯*A*	*D*—H⋯*A*
C12—H12*C*⋯O2^i^	0.96	2.51	3.315 (3)	142
C14—H14⋯O2^i^	0.93	2.57	3.298 (3)	135

Symmetry code: (i) 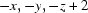.

**Fig. 1.**
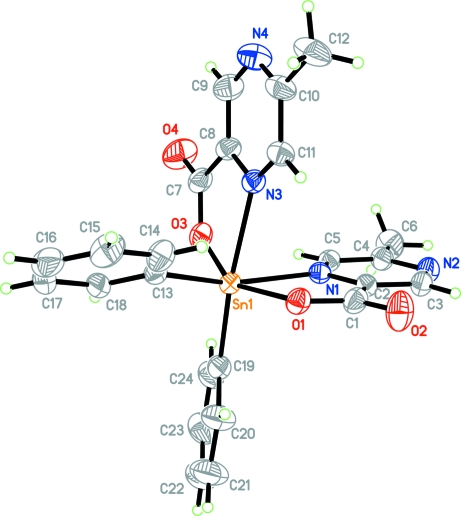
The molecular structure of the title molecule, with the atom-numbering scheme. Displacement ellipsoids are drawn at the 30% probability level.

**Fig. 2.**
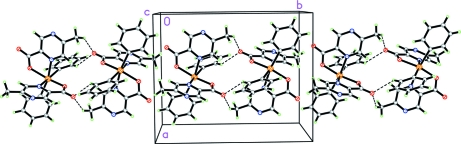
A partial packing diagram of (I). Hydrogen bonds are shown as dashed lines.

## supplementary crystallographic information

### Comment

Self-assembled organotin derivatives of carboxylic acid ligands have been extensively studied due to their biological activities (Gielen *et al.*, 1988). 5-methylpyrazine-2-carboxylic acid is a good bridging ligand that can sometimes be used to generate unexpected and interesting coordination polymers, and small changes in experimental conditions can lead to very different architectures (Ma *et al.*,  2004).

The molecule of the title compound, (I), (Fig. 1) consists of two phenyl and two (5-methylpyrazine-2-carboxylate) groups bonded to the Sn atom and has a monomeric structure. The two O and the one N atoms of the two 2-methylpyrazine -5-carboxylate ligands and the one C atom of the one phenyl group in the equatorial plane around the Sn atom form a distorted square-planar arrangement, while the distorted octahedral coordination is completed by the one N atom of the one 5-methylpyrazine-2-carboxylate ligand and the one C atom of the other phenyl group in the axial positions (Table 1 and Fig. 1). The Sn1-O1 [2.086 (4) Å] and Sn1-O3 [2.091 (4) Å] bonds are much shorter than the van der Waal's sum of 4.0 Å (Vollano *et al.*,  1984).

In the crystal structure, intermolecular C-H···O hydrogen bonds (Table 2) link the molecules into centrosymmetric dimers (Fig. 2), in which they may be effective in the stabilization of the structure.

### Experimental

For the preparation of the title compound, a mixture of diphenyltin dichloride (344 mg, 1.0 mmol), 5-methylpyrazine-2-carboxylic acid (276 mg, 2.0 mmol) and sodium ethoxide (136 mg, 2.0 mmol) in ethanol (80 ml) was heated under reflux for 12 h at 303 K. The resulting clear solution was evaporated under vacuum and the product recrystallized from a mixture of methanol to yield colorless, block-like crystals of (I) (yield; 377 mg, 69%, m.p. 459 K). Analysis, calculated for (I): C 52.68, H, 3.68; N 10.24%; found: C 52.96, H 3.87, N, 10.11%.

### Refinement

H atoms were positioned geometrically, with C-H = 0.93 and 0.96 Å for aromatic and methyl H, respectively, and constrained to ride on their parent atoms with U_iso_(H) = xU_eq_(C), where x = 1.5 for methyl H, and x = 1.2 for aromatic H atoms.

### Crystal data

**Table:**

[Sn(C_6_H_5_)_2_(C_6_H_5_N_2_O_2_)_2_]	*F*_000_ = 1096
*M**_r_* = 547.13	*D*_x_ = 1.538 Mg m^−^^3^
Monoclinic, *P*2_1_/*n*	Mo *K*α radiation λ = 0.71073 Å
Hall symbol: -P 2yn	Cell parameters from 3659 reflections
*a* = 12.030 (4) Å	θ = 2.2–24.0º
*b* = 14.658 (5) Å	µ = 1.12 mm^−^^1^
*c* = 13.409 (5) Å	*T* = 298 (2) K
β = 91.872 (4)º	Block, colorless
*V* = 2363.2 (14) Å^3^	0.45 × 0.43 × 0.18 mm
*Z* = 4	

### Data collection

**Table:**

Bruker SMART CCD area-detector diffractometer	4166 independent reflections
Radiation source: fine-focus sealed tube	2732 reflections with *I* > 2σ(*I*)
Monochromator: graphite	*R*_int_ = 0.039
*T* = 298(2) K	θ_max_ = 25.0º
φ and ω scans	θ_min_ = 2.1º
Absorption correction: multi-scan(SADABS; Sheldrick, 1996)	*h* = −12→14
*T*_min_ = 0.633, *T*_max_ = 0.824	*k* = −17→14
12010 measured reflections	*l* = −15→15

### Refinement

**Table:**

Refinement on *F*^2^	Secondary atom site location: difference Fourier map
Least-squares matrix: full	Hydrogen site location: inferred from neighbouring sites
*R*[*F*^2^ > 2σ(*F*^2^)] = 0.035	H-atom parameters constrained
*wR*(*F*^2^) = 0.132	*w* = 1/[σ^2^(*F*_o_^2^) + (0.066*P*)^2^ + 2.0808*P*] where *P* = (*F*_o_^2^ + 2*F*_c_^2^)/3
*S* = 1.05	(Δ/σ)_max_ < 0.001
4166 reflections	Δρ_max_ = 0.56 e Å^−^^3^
298 parameters	Δρ_min_ = −0.46 e Å^−^^3^
Primary atom site location: structure-invariant direct methods	Extinction correction: none

### Special details

**Table:**

Geometry. All e.s.d.'s (except the e.s.d. in the dihedral angle between two l.s. planes) are estimated using the full covariance matrix. The cell e.s.d.'s are taken into account individually in the estimation of e.s.d.'s in distances, angles and torsion angles; correlations between e.s.d.'s in cell parameters are only used when they are defined by crystal symmetry. An approximate (isotropic) treatment of cell e.s.d.'s is used for estimating e.s.d.'s involving l.s. planes.
Refinement. Refinement of F^2^ against ALL reflections. The weighted R-factor wR and goodness of fit S are based on F^2^, conventional R-factors R are based on F, with F set to zero for negative F^2^. The threshold expression of F^2^ > 2sigma(F^2^) is used only for calculating R-factors(gt) etc. and is not relevant to the choice of reflections for refinement. R-factors based on F^2^ are statistically about twice as large as those based on F, and R- factors based on ALL data will be even larger.

### Fractional atomic coordinates and isotropic or equivalent isotropic displacement parameters (Å^2^)

**Table:**

	*x*	*y*	*z*	*U*_iso_*/*U*_eq_	
Sn1	0.03995 (3)	0.25533 (2)	0.90574 (3)	0.04317 (17)	
N1	0.0859 (4)	0.2891 (3)	1.0739 (3)	0.0440 (11)	
N2	0.1584 (4)	0.3005 (4)	1.2707 (4)	0.0611 (13)	
N3	−0.1259 (4)	0.2163 (3)	0.9852 (3)	0.0473 (11)	
N4	−0.3408 (5)	0.1915 (4)	1.0421 (5)	0.0788 (17)	
O1	0.0994 (3)	0.1346 (2)	0.9701 (3)	0.0517 (10)	
O2	0.1706 (4)	0.0621 (3)	1.1024 (4)	0.0853 (15)	
O3	−0.0532 (3)	0.3752 (2)	0.9163 (3)	0.0545 (10)	
O4	−0.2151 (4)	0.4402 (3)	0.9433 (4)	0.0946 (16)	
C1	0.1354 (5)	0.1303 (4)	1.0620 (5)	0.0557 (15)	
C2	0.1298 (5)	0.2175 (4)	1.1213 (4)	0.0450 (13)	
C3	0.1662 (5)	0.2249 (4)	1.2196 (5)	0.0556 (15)	
H3	0.1976	0.1740	1.2509	0.067*	
C4	0.1149 (5)	0.3732 (4)	1.2234 (4)	0.0541 (15)	
C5	0.0804 (5)	0.3667 (4)	1.1242 (4)	0.0488 (14)	
H5	0.0525	0.4184	1.0918	0.059*	
C6	0.1053 (6)	0.4589 (4)	1.2806 (5)	0.082 (2)	
H6A	0.1337	0.4496	1.3476	0.122*	
H6B	0.0286	0.4767	1.2820	0.122*	
H6C	0.1474	0.5060	1.2494	0.122*	
C7	−0.1548 (5)	0.3744 (4)	0.9450 (5)	0.0570 (16)	
C8	−0.1966 (5)	0.2842 (4)	0.9824 (4)	0.0513 (14)	
C9	−0.3049 (6)	0.2725 (5)	1.0106 (6)	0.075 (2)	
H9	−0.3537	0.3217	1.0078	0.090*	
C10	−0.2715 (6)	0.1230 (5)	1.0447 (5)	0.0649 (18)	
C11	−0.1606 (5)	0.1348 (4)	1.0159 (4)	0.0536 (15)	
H11	−0.1117	0.0857	1.0183	0.064*	
C12	−0.3116 (6)	0.0326 (5)	1.0790 (6)	0.091 (2)	
H12A	−0.3885	0.0371	1.0953	0.136*	
H12B	−0.2685	0.0138	1.1369	0.136*	
H12C	−0.3037	−0.0115	1.0267	0.136*	
C13	−0.0319 (4)	0.1966 (4)	0.7745 (4)	0.0469 (13)	
C14	−0.0653 (5)	0.1070 (4)	0.7688 (5)	0.0665 (17)	
H14	−0.0584	0.0704	0.8253	0.080*	
C15	−0.1086 (6)	0.0704 (5)	0.6821 (6)	0.080 (2)	
H15	−0.1298	0.0094	0.6801	0.096*	
C16	−0.1207 (6)	0.1231 (7)	0.5990 (6)	0.088 (2)	
H16	−0.1514	0.0983	0.5405	0.105*	
C17	−0.0877 (6)	0.2125 (7)	0.6012 (5)	0.083 (2)	
H17	−0.0953	0.2487	0.5444	0.100*	
C18	−0.0427 (5)	0.2482 (4)	0.6896 (5)	0.0645 (18)	
H18	−0.0194	0.3087	0.6911	0.077*	
C19	0.1901 (5)	0.3181 (4)	0.8612 (4)	0.0556 (15)	
C20	0.2684 (7)	0.2621 (5)	0.8185 (7)	0.084 (2)	
H20	0.2541	0.1998	0.8142	0.101*	
C21	0.3653 (7)	0.2945 (8)	0.7828 (7)	0.111 (3)	
H21	0.4156	0.2557	0.7529	0.134*	
C22	0.3867 (8)	0.3866 (7)	0.7921 (6)	0.104 (2)	
H22	0.4537	0.4096	0.7701	0.124*	
C23	0.3137 (7)	0.4438 (6)	0.8320 (6)	0.090 (2)	
H23	0.3286	0.5060	0.8365	0.108*	
C24	0.2133 (6)	0.4077 (5)	0.8671 (5)	0.0750 (19)	
H24	0.1620	0.4469	0.8950	0.090*	

### Atomic displacement parameters (Å^2^)

**Table:**

	*U*^11^	*U*^22^	*U*^33^	*U*^12^	*U*^13^	*U*^23^
Sn1	0.0388 (2)	0.0483 (3)	0.0423 (2)	0.00011 (17)	−0.00113 (16)	0.00171 (17)
N1	0.040 (3)	0.046 (2)	0.046 (3)	−0.006 (2)	0.001 (2)	0.003 (2)
N2	0.058 (3)	0.075 (4)	0.050 (3)	−0.005 (3)	−0.006 (2)	0.004 (3)
N3	0.045 (3)	0.049 (3)	0.048 (3)	0.000 (2)	−0.001 (2)	−0.003 (2)
N4	0.068 (4)	0.081 (4)	0.090 (4)	0.002 (3)	0.025 (3)	−0.005 (3)
O1	0.054 (2)	0.047 (2)	0.055 (2)	0.0109 (17)	−0.0018 (19)	−0.0026 (17)
O2	0.121 (4)	0.052 (3)	0.081 (3)	0.018 (3)	−0.015 (3)	0.012 (2)
O3	0.058 (3)	0.046 (2)	0.059 (2)	0.0037 (18)	−0.009 (2)	0.0066 (17)
O4	0.080 (4)	0.067 (3)	0.136 (5)	0.030 (3)	0.001 (3)	0.006 (3)
C1	0.051 (4)	0.053 (4)	0.063 (4)	0.004 (3)	−0.005 (3)	0.011 (3)
C2	0.044 (3)	0.049 (3)	0.042 (3)	−0.003 (3)	0.002 (3)	0.006 (3)
C3	0.051 (4)	0.063 (4)	0.052 (4)	0.000 (3)	−0.006 (3)	0.011 (3)
C4	0.053 (4)	0.063 (4)	0.046 (3)	−0.008 (3)	−0.003 (3)	−0.006 (3)
C5	0.044 (3)	0.048 (3)	0.053 (3)	−0.004 (2)	−0.001 (3)	0.000 (3)
C6	0.096 (6)	0.081 (5)	0.068 (5)	−0.004 (4)	−0.004 (4)	−0.023 (4)
C7	0.053 (4)	0.057 (4)	0.061 (4)	0.015 (3)	−0.005 (3)	−0.003 (3)
C8	0.044 (4)	0.059 (3)	0.051 (3)	0.007 (3)	0.004 (3)	−0.015 (3)
C9	0.060 (5)	0.080 (5)	0.085 (5)	0.023 (4)	0.008 (4)	−0.003 (4)
C10	0.064 (5)	0.075 (5)	0.057 (4)	−0.014 (4)	0.018 (3)	−0.013 (3)
C11	0.054 (4)	0.053 (4)	0.055 (4)	−0.003 (3)	0.009 (3)	−0.005 (3)
C12	0.087 (6)	0.098 (6)	0.090 (6)	−0.033 (4)	0.032 (4)	0.002 (4)
C13	0.039 (3)	0.057 (4)	0.045 (3)	−0.005 (3)	−0.001 (3)	−0.003 (3)
C14	0.065 (4)	0.065 (4)	0.069 (4)	−0.005 (3)	−0.015 (3)	−0.007 (3)
C15	0.074 (5)	0.071 (5)	0.095 (6)	−0.006 (4)	−0.017 (4)	−0.027 (4)
C16	0.059 (5)	0.132 (8)	0.072 (5)	−0.004 (5)	−0.004 (4)	−0.036 (5)
C17	0.062 (5)	0.143 (7)	0.044 (4)	−0.010 (5)	−0.001 (3)	0.004 (4)
C18	0.051 (4)	0.095 (5)	0.047 (4)	−0.015 (3)	0.002 (3)	0.006 (3)
C19	0.051 (3)	0.066 (4)	0.050 (3)	−0.007 (3)	−0.001 (3)	0.001 (3)
C20	0.069 (5)	0.089 (5)	0.095 (5)	−0.006 (4)	0.026 (4)	0.003 (4)
C21	0.077 (5)	0.147 (6)	0.112 (6)	−0.008 (5)	0.042 (4)	−0.001 (5)
C22	0.082 (5)	0.130 (6)	0.099 (5)	−0.030 (5)	0.015 (4)	0.003 (5)
C23	0.097 (5)	0.096 (5)	0.077 (5)	−0.038 (4)	0.006 (4)	0.002 (4)
C24	0.073 (4)	0.089 (5)	0.063 (4)	−0.023 (4)	0.003 (3)	0.007 (3)

### Geometric parameters (Å, °)

**Table:**

Sn1—O1	2.086 (4)	C9—H9	0.9300
Sn1—O3	2.091 (4)	C10—C11	1.411 (8)
Sn1—C13	2.117 (5)	C10—C12	1.488 (9)
Sn1—C19	2.130 (6)	C11—H11	0.9300
Sn1—N1	2.357 (4)	C12—H12A	0.9600
Sn1—N3	2.363 (5)	C12—H12B	0.9600
N1—C5	1.324 (7)	C12—H12C	0.9600
N1—C2	1.328 (7)	C13—C18	1.370 (8)
N2—C3	1.308 (8)	C13—C14	1.375 (8)
N2—C4	1.338 (7)	C14—C15	1.368 (9)
N3—C8	1.309 (7)	C14—H14	0.9300
N3—C11	1.335 (7)	C15—C16	1.360 (10)
N4—C10	1.305 (8)	C15—H15	0.9300
N4—C9	1.336 (9)	C16—C17	1.370 (10)
O1—C1	1.294 (7)	C16—H16	0.9300
O2—C1	1.207 (6)	C17—C18	1.389 (10)
O3—C7	1.292 (7)	C17—H17	0.9300
O4—C7	1.207 (7)	C18—H18	0.9300
C1—C2	1.508 (8)	C19—C24	1.344 (9)
C2—C3	1.379 (8)	C19—C20	1.387 (9)
C3—H3	0.9300	C20—C21	1.361 (11)
C4—C5	1.384 (7)	C20—H20	0.9300
C4—C6	1.477 (8)	C21—C22	1.380 (12)
C5—H5	0.9300	C21—H21	0.9300
C6—H6A	0.9600	C22—C23	1.339 (11)
C6—H6B	0.9600	C22—H22	0.9300
C6—H6C	0.9600	C23—C24	1.413 (9)
C7—C8	1.506 (9)	C23—H23	0.9300
C8—C9	1.379 (9)	C24—H24	0.9300
			
O1—Sn1—O3	149.56 (15)	N4—C9—C8	121.0 (6)
O1—Sn1—C13	97.14 (18)	N4—C9—H9	119.5
O3—Sn1—C13	101.11 (18)	C8—C9—H9	119.5
O1—Sn1—C19	101.5 (2)	N4—C10—C11	120.5 (6)
O3—Sn1—C19	96.7 (2)	N4—C10—C12	118.7 (6)
C13—Sn1—C19	105.6 (2)	C11—C10—C12	120.8 (6)
O1—Sn1—N1	73.49 (15)	N3—C11—C10	120.2 (6)
O3—Sn1—N1	82.40 (15)	N3—C11—H11	119.9
C13—Sn1—N1	163.15 (18)	C10—C11—H11	119.9
C19—Sn1—N1	90.17 (18)	C10—C12—H12A	109.5
O1—Sn1—N3	83.74 (16)	C10—C12—H12B	109.5
O3—Sn1—N3	73.16 (16)	H12A—C12—H12B	109.5
C13—Sn1—N3	87.08 (18)	C10—C12—H12C	109.5
C19—Sn1—N3	165.3 (2)	H12A—C12—H12C	109.5
N1—Sn1—N3	78.12 (15)	H12B—C12—H12C	109.5
C5—N1—C2	117.4 (5)	C18—C13—C14	117.5 (6)
C5—N1—Sn1	130.8 (4)	C18—C13—Sn1	119.5 (4)
C2—N1—Sn1	111.6 (4)	C14—C13—Sn1	123.0 (4)
C3—N2—C4	117.5 (5)	C15—C14—C13	121.7 (7)
C8—N3—C11	118.7 (5)	C15—C14—H14	119.2
C8—N3—Sn1	111.1 (4)	C13—C14—H14	119.2
C11—N3—Sn1	129.3 (4)	C16—C15—C14	120.1 (7)
C10—N4—C9	118.7 (6)	C16—C15—H15	119.9
C1—O1—Sn1	122.3 (3)	C14—C15—H15	119.9
C7—O3—Sn1	121.8 (3)	C15—C16—C17	120.2 (7)
O2—C1—O1	124.7 (6)	C15—C16—H16	119.9
O2—C1—C2	119.0 (6)	C17—C16—H16	119.9
O1—C1—C2	116.2 (5)	C16—C17—C18	118.9 (7)
N1—C2—C3	120.2 (5)	C16—C17—H17	120.5
N1—C2—C1	116.2 (5)	C18—C17—H17	120.5
C3—C2—C1	123.5 (5)	C13—C18—C17	121.7 (7)
N2—C3—C2	122.7 (6)	C13—C18—H18	119.2
N2—C3—H3	118.6	C17—C18—H18	119.2
C2—C3—H3	118.6	C24—C19—C20	117.4 (6)
N2—C4—C5	120.0 (5)	C24—C19—Sn1	125.6 (5)
N2—C4—C6	117.9 (5)	C20—C19—Sn1	116.9 (5)
C5—C4—C6	122.1 (6)	C21—C20—C19	122.6 (8)
N1—C5—C4	122.0 (5)	C21—C20—H20	118.7
N1—C5—H5	119.0	C19—C20—H20	118.7
C4—C5—H5	119.0	C20—C21—C22	118.0 (9)
C4—C6—H6A	109.5	C20—C21—H21	121.0
C4—C6—H6B	109.5	C22—C21—H21	121.0
H6A—C6—H6B	109.5	C23—C22—C21	121.8 (9)
C4—C6—H6C	109.5	C23—C22—H22	119.1
H6A—C6—H6C	109.5	C21—C22—H22	119.1
H6B—C6—H6C	109.5	C22—C23—C24	118.5 (8)
O4—C7—O3	124.2 (6)	C22—C23—H23	120.7
O4—C7—C8	120.0 (6)	C24—C23—H23	120.7
O3—C7—C8	115.9 (5)	C19—C24—C23	121.6 (7)
N3—C8—C9	121.1 (6)	C19—C24—H24	119.2
N3—C8—C7	116.9 (5)	C23—C24—H24	119.2
C9—C8—C7	122.0 (6)		

### Hydrogen-bond geometry (Å, °)

**Table:**

*D*—H···*A*	*D*—H	H···*A*	*D*···*A*	*D*—H···*A*
C12—H12C···O2^i^	0.96	2.51	3.315 (3)	142
C14—H14···O2^i^	0.93	2.57	3.298 (3)	135

Symmetry codes: (i) −*x*, −*y*, −*z*+2.

## References

[bb1] Bruker (1998). *SMART* and *SAINT* Bruker AXS Inc., Madison, Wisconsin, USA.

[bb2] Gielen, M., Vanbellinghen, C., Gelan, J. & Willem, R. (1988). *Bull. Soc. Chim. Belg.* **97**, 873–876.

[bb3] Ma, C. L., Han, Y. F., Zhang, R. F. & Wang, D. Q. (2004). *Dalton Trans.* pp. 1832–1840. 10.1039/b404477k 15381988

[bb4] Sheldrick, G. M. (1996). *SADABS* University of Göttingen, Germany.

[bb5] Sheldrick, G. M. (2008). *Acta Cryst.* A**64**, 112–122. 10.1107/S0108767307043930 18156677

[bb6] Vollano, J. F., Day, R. O. & Holmes, R. R. (1984). *Organometallics*, **3**, 745–750.

